# Scientific Items

**Published:** 1879-09-20

**Authors:** 


### SCIENTIFIC ITEMS.

**The Sand Blast.**—Among the wonderful and useful inventions of the times is the common sand blast. Suppose you desire to letter a piece of marble for a gravestone; you cover the stone with a sheet of wax no thicker than a wafer, then cut in the wax the name, date, etc., leaving the marble exposed. Now pass it under the blast, and the wax will not be injured at all, but the sand will cut letters deep into the stone.

Or if you desire raised letters, a flower or other emblem, cut the letters, flowers, etc., in wax, and stick them upon the stone; then pass the stone under the blast and the sand will cut it away. Remove the wax and you have the raised letters.

Take a piece of French plate glass, say two feet by six, and cover it with fine lace, pass it under the blast, and not a thread of the lace will be injured, but the sand will cut deep into the glass wherever it is not covered with lace.

Now remove the lace and you have every delicate and beautiful figure raised upon the glass.

In this way beautiful figures of all kinds are cut in glass, and at a small expense. The workmen can hold their hands under the blast without harm, even when it is rapidly cutting away the hardest glass, iron or stone, but they must look out for finger nails, for they will be whittled off right hastily. If they put on steel thimbles to protect the nails, it will do little good, for the sand will soon whittle them away, but if they wrap a piece of soft cotton around them they are safe.

You will at once see the philosophy of it. The sand whittles away and destroys any hard substance, even glass, but does not affect substances that are soft and yielding like wax, cotton or fine lace, or even the human hand.—*Portland Argus.*

**The Electric Light.**—Prof. Cohn, of Breslau, has been lately making experiments with the electric light on the eyes of a number of persons, for the purpose of testing its influence on visual perception and the sensation of color. He has found that letters, spots and colors are perceived at a much greater distance through the medium of electric light than by day or gaslight.

The sensation of yellow was increased sixty-fold compared to daylight; of red six-fold; and of green and blue, about two-fold. Eyes that could only with difficulty perceive and distinguish colors by daylight or gas-light were much aided by the electric light, and the visual perception was also much strengthened.

Prof. Cohn concludes from this fact that electric light would prove exceedingly useful in places where it is desirable that signals should be seen at a great distance.

The engine used was Gramme's electro-magnetic apparatus, which rotates six hundred times in a minute.—*Brit. Med. Journal.*

**Medicines by Galvanism.**—Ever since the day of Sir Humphry Davy, the possibility of introducing medicines into living bodies has been thought to be possible, and perhaps even done. At least certain salts in solution, under the influence of an electric current, have been decomposed, and their elements found at the positive and negative poles respectively, having been made to pass through living tissues which composed part of a closed circuit of the galvanic arrangement. But to deposit medicines is somewhat different. Instead of going through they must be dropped on the way. To do this appears to have been thought a difficulty.

Herman Munk has discovered that the failure is because the current was in only one direction. He found that if a moist, porous body, between liquids of various conductivity, be traversed by the current, the speed of the conveyance of the liquid into this body rapidly diminishes and becomes soon at zero. If, however, the current is reversed after a short interval, the liquid enters anew from the now positive electrode. By repeating this alternation, large quantities of the liquid can be introduced. In this manner Mr. Munk has introduced fatal quantities of strychnia solution through the unbroken skin of dogs, and has introduced quinine and iodide of potassium into his own arm in such quantities as to be readily detected in the excreta. The essential points, therefore, in such operations are that the liquid substance be placed at both electrodes, and that the direction of the current be frequently reversed.—*Drug. Circular.*

**Fecundity and Sexuality.**—At a recent meeting of the *Societe de Biologie*, M. Delaunay read a paper upon this subject, in which he asserted that fecundity was in inverse proportion to the elevation of the race. Thus, the colored races are more fertile than the white, and, of the latter, those least advanced—*e.g.*, Russia, Spain, Ireland, Italy,—are more fertile than the highly civilized, as France and Switzerland. It has been said that the relative sterility of France is voluntary. M. Delaunay protested against this accusation. Fecundity diminishes in a race in proportion to its evolution. The educated classes and citizens are less fertile than country-people. The law of Malthus is thus shown to be erroneous.

Inferior races show more females; superior races more males. After thirty-five years a man begets more girls than boys. The strong have boys; the weak girls. More girls are generated in warm climates and during the summer, and more boys in cold climates and during the winter.

In commenting upon the statements of M. Delaunay, M. Gallipe criticised them as not being based upon satisfactory statistics. He called attention to a fact which falsified M. Delaunay's conclusions—namely, that England, a country in the front rank of civilization, shows remarkable fecundity. [Perhaps the most interesting part of M. Delaunay's paper is the protest against the generally received opinion that the sterility of the French is voluntary.]—*Le Progres Medical.*

**Sonometry by an Electrical Induction Balance.**—Prof. D. E. Hughes recently read a most instructive paper before the Royal Society on the subject of accurately adjusting currents of electricity so as to completely neutralize each other, coming from opposite directions so as to strike an exact balance. The apparatus is simple, inexpensive and conveniently portable. Prof. Hughes calls it the sonometer, or audiometer, and it is intended for conducting aural investigation with absolute accuracy, a line of inquiry hitherto limited, for the want of such an instrument.

